# Outcomes of off-label drug uses in hospitals: a multicentric prospective study

**DOI:** 10.1007/s00228-014-1746-2

**Published:** 2014-09-09

**Authors:** I. Danés, A. Agustí, A. Vallano, C. Alerany, J. Martínez, J. A. Bosch, A. Ferrer, L. Gratacós, A. Pérez, M. Olmo, S. M. Cano Marron, A. Valderrama, X. Bonafont

**Affiliations:** 1 Clinical Pharmacology Service, Fundació Institut Català de Farmacologia. Hospital Universitari Vall d’Hebron, Department of Pharmacology, Therapeutics and Toxicology, Universitat Autònoma de Barcelona, Passeig Vall d’Hebron, 119-129 Barcelona, Spain; 2 Clinical Pharmacology Service, Hospital Universitari de Bellvitge, IDIBELL, Department of Pathology and Experimental Therapeutics, Universitat de Barcelona, Hospitalet de Llobregat, Barcelona, Spain; 3 Pharmacy Service, Hospital Universitari Vall d’Hebron, Barcelona, Spain; 4 Medical Direction, Department of Internal Medicine, Hospital Universitari Vall d’Hebron, Universitat Autònoma de Barcelona, Barcelona, Spain; 5 Pharmacy Service, Hospital Universitari de Bellvitge, IDIBELL, Hospitalet de Llobregat, Barcelona, Spain; 6 Pharmacy Service, Hospital Universitari Josep Trueta, Girona, Spain; 7 Pharmacy Service, Hospital Universitari Arnau de Vilanova, Lleida, Spain; 8 Clinical Pharmacology Service, Hospital Universitari Germans Trias i Pujol, Badalona, Spain; 9 Pharmacy Service, Hospital Universitari Germans Trias i Pujol, Badalona, Spain

**Keywords:** Off-label use, Drug therapy, Efficiency, Rituximab, Omalizumab, Botulinum toxin, Pharmacy and therapeutics committees

## Abstract

**Purpose:**

The study aims to assess the clinical evidence, outcome and cost of off-label use of medicines in the hospital setting.

**Methods:**

A multicentric prospective cohort study of patients treated with off-label medicines was carried out in five tertiary hospitals from May 2011 to May 2012. Information on clinical characteristics of patients, drugs, outcomes and costs was collected. Patients were followed up to 6 months, and information was assessed by reviewing clinical records and interviewing physicians.

**Results:**

A total of 226 patients were included. The median (interquartile range (IQR)) age of patients was 46 (33–62) years; 59 % were women. Patients had received a median of three previous treatments, and a lack of response (or suboptimal) was the main reason for off-label use (72.1 %). A total of 232 off-label medicines were administered for 102 different indications. The most frequent medicines were rituximab (49; 21.1 %), botulinum toxin (25; 10.7 %) and omalizumab (14; 6.0 %). In 117 (51.8 %) cases, the level of clinical evidence for their use was low. A partial clinical response was observed in 82 patients (36.3 %), complete response in 71 (31.4 %) and stabilization in 11 (4.9 %). A total of 58 (26.5 %) patients had adverse effects, which in 11 (4.9 %) were severe. The median (IQR) cost per patient was €2,943.07 (541.9–5,872.54).

**Conclusions:**

There was a high variability of off-label medicines and indications. Although the clinical evidence of off-label medicines was often low, clinical response was observed in many patients with previous multiple treatment failure, but at the expense of some adverse effects and a high cost. Registers of patients would be helpful for clinical decisions, although clinical trials are needed.

**Electronic supplementary material:**

The online version of this article (doi:10.1007/s00228-014-1746-2) contains supplementary material, which is available to authorized users.

## Introduction

Off-label medicine use includes the prescription of a medicine for an indication, a route of administration or a patient group that is not approved in the summary product characteristics [1]. The off-label use of medicines is a common and widespread clinical practice worldwide [2, 3]. However, the use of medicines outside the approved clinical indications may lead to several problems. Evidence on the use of these medicines in unapproved indications is often scarce, and doctors have little information on how to use them. In addition, off-label use of medicines can cause adverse effects and the risk may outweigh the potential benefits. Furthermore, ethical and legal issues related to the commercial promotion of off-label use of these medications have also been raised [4–6].

Since 2009, a new Spanish legislation regulates and classifies the availability of drug use in special situations: the use of medicines in unapproved conditions, the compassionate use of investigational medicines and the use of medicines not marketed in the country [7]. Currently, only a doctor’s report to justify the use of the off-label medicines and the patient’s informed consent are required. Nevertheless, the widespread use of these drugs may often increase spending on drugs, especially in the hospital setting. In order to avoid unwarranted risks and cost of drugs with limited data on their efficacy, the Catalan Health Service has put internal procedures in place [8]. This regulation states that the drug and therapeutics committees of each hospital needs to perform an evaluation of all cases of drug use in special situations, and the medical director of each hospital must give individual authorisation for each patient.

Several studies have evaluated the use of off-label medicines, but they have often focused on specific groups of drugs or medicines, such as anticancer drugs [9–11] or rituximab [12, 13], or on specific populations, such as children [14–17]. However, very few studies have evaluated the clinical outcomes of off-label medicines in terms of effectiveness and safety as well as the associated costs [12, 18, 19]. The aim of our study was to assess the clinical and economic outcomes and the clinical evidence for off-label use of medicines in the hospital setting.

## Methods

A prospective longitudinal study of patients treated with off-label drugs was carried out in five public hospitals belonging to the Catalan Institute of Health for a period of 1 year (from 19th May 2011 to 19th May 2012). Requests for drugs use in special situations, taking into account the current Spanish legislation, received in the pharmacy services of the hospitals during the study period were identified. All requests for off-label uses were included, and those for compassionate use of investigational drugs and for unauthorized drugs in Spain (if requested for conditions approved in other countries) were excluded. In addition, those for off-label drug use that were not authorized by hospital medical directors, those for which patient informed consent was not obtained or those in whom medicines were not finally administered, were also excluded. A prospective review of the application forms of off-label drug use and the patients’ electronic medical records was conducted to obtain information on patients’ demographic characteristics, morbidity (clinical, biological and other complementary explorations), previous and concurrent drug uses for the target disease, the requested drug and dosages, the clinical indications and the reasons for the requested off-label drug use, and the clinical outcomes (effectiveness and adverse drug effects). Patients were followed for a period of 6 months after starting off-label drug treatment (or until the end of treatment in cases of an acute disease), and the clinical outcomes were assessed by reviewing electronic clinical records and interviewing physicians responsible for the patient’s care.

Drugs were classified according to the ATC classification, and The International Classification of Diseases, ninth edition (ICD-9), was used to classify medical indication for off-label drug use. Off-label drug use condition was rated as an unapproved indication, unapproved condition (population, route or other) or both. The reasons for requesting the off-label drug use were categorized as the following: lack of clinical response to previous treatments (or suboptimal), intolerance or contraindications to the alternatives or other reasons such as unavailability of approved drugs for that indication/condition or preferred in that patient to the alternatives due to clinical or logistical reasons.

A review of published evidence for every drug use in each clinical indication was performed searching for information on the PubMed database. In addition, a search looking at ongoing clinical trials for every drug use in each clinical indication was conducted in clinicaltrials.gov register [20]. The Oxford Centre for Evidence Based Medicine criteria was used to classify the available evidence found for each requested drug in each indication [21]. The level of evidence was pooled into two categories: the high level category that included the 1a to 2c categories (mainly randomized clinical trials or cohort studies) and the low level that included the 3 to 5 categories (mainly case-control studies, series of cases, cases and expert opinions).

The clinical responses to off-label use of drugs were classified as complete response (CR), partial response (PR), stabilization (S) and no response (NR) taking into account different parameters of efficacy for each disease. For example, the criteria used in the more common diseases are specified. For botulinum toxin in anal fissure, the healing of the lesion was considered CR and its persistence without symptoms was considered PR. In patients with esophageal achalasia, clinical criteria were also used: CR if the patients were able to eat without dysphagia and PR if they felt some improvement. CR to omalizumab in chronic urticaria was considered if corticosteroids could be withdrawn and the patient was asymptomatic or had minimal symptoms; other minor improvements were classified as PR. To assess the response to rituximab in patients with organ transplant rejection, anatomopathological criteria were used, and in pemphigus and myasthenia, clinical criteria were used (resolution was considered CR, and improvement PR). In patients with systemic lupus erythematosus, the symptoms and scores of disease activity were taken into account [CR, Systemic Lupus Erythematosus Disease Activity Index (SLEDAI) of ≤4 or clinical remission; PR, improvement of ≥50 % in SLEDAI]. For human-unspecific immunoglobulins in immune encephalitis, clinical criteria (resolution or improvement) were considered.

Adverse drug events were assessed by clinical pharmacologists and/or pharmacists trained in using the methods and the algorithm of the Spanish Pharmacovigilance System.

The actual sale price of medicines paid by participant hospitals was taken into account in the analysis of the cost of treatments. The total cost per patient was calculated according to the duration of treatment up to a maximum of 6 months.

The study was conducted in accordance with the international ethics recommendations and according to the Spanish post-authorization studies legislation. The study protocol was approved by the ethics committees of clinical investigation in each participating hospital.

Statistical analysis of categorical and continuous variables was made by means of the distribution of frequencies, proportions, means, standard deviation (SD) and median and interquartile range (IQR). Statistical differences were evaluated using the chi-square test and Student’s *t* test. Significance was set at a level of 0.05 and was two-tailed. The statistical analysis was performed using IBM SPSS Statistics version 20 statistical package (IBM corp., NY, USA).

## Results

**Fig. 1:**
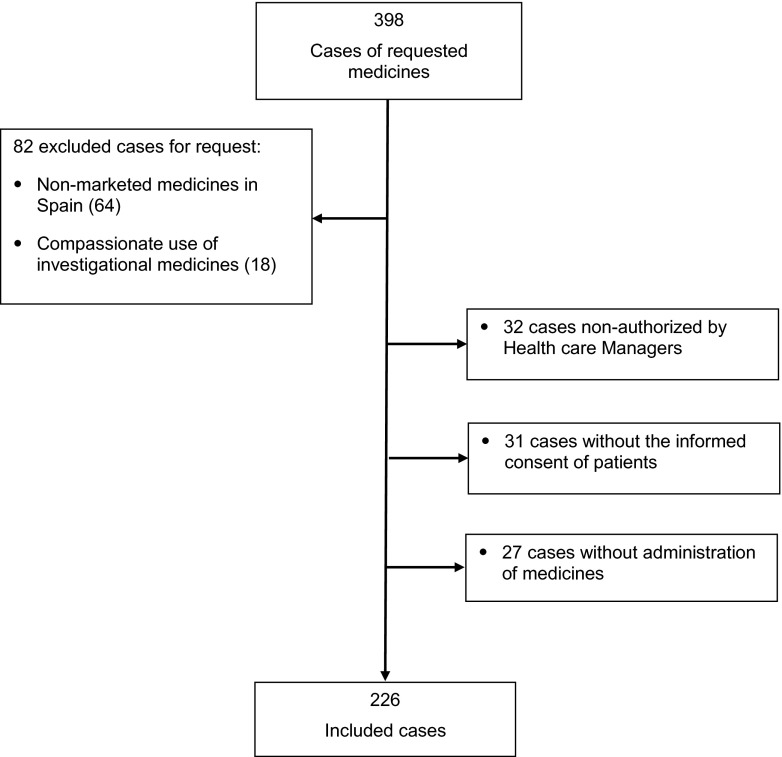
Flowchart of the included patients in the study

A total of 398 requests for treating the corresponding patients were received, and 226 were included in the study (each participating hospital contributed with 85 (37.6 %), 56 (24.8 %), 42 (18.6 %), 28 (12.4 %) and 15 (6.6 %) cases, respectively). The reasons for the exclusions are shown in Fig. 1. The characteristics of patients treated are shown in Table 1. The median age (IQR) of treated patients was 46 (33–62) years and 59 % were women. The patients involved had received on average three previous treatments for the target diseases, and in 163 cases (72.1 %), lack of response to previous treatments (or suboptimal) was the main reason for requesting the off-label drug use. In 90.3 % of cases, the requested off-label drugs were for an unapproved indication. Clinical services that most frequently requested off-label drug use were gastroenterology, internal medicine and neurology.

**Table 1:** Clinical characteristics of patients and requests

Characteristics	Patients (*N* = 226)
Age (median, IQR) years	46 (33–62)
< 18 years (%)	28 (12.4)
18–64 years (%)	147 (65.0)
≥65 years (%)	51 (22.6)
Gender (%)	
Female	133 (59)
Male	93 (41)
Morbidity (%)	
Arterial hypertension (%)	58 (25.7)
Hyperlipidemia (%)	32 (14.2)
Diabetes (%)	25 (11.1)
Chronic renal failure (%)	14 (6.2)
Coronary heart disease	13 (5.7)
Heart failure (%)	3 (1.3)
Previous treatments for the target diseases (median, IQR)	3 (2–5)
Clinical services (%)	
Gastroenterology	33 (14.6)
Internal medicine	30 (13.3)
Neurology	28 (12.4)
Paediatrics	24 (10.6)
Oncology	14 (6.2)
Allergy	14 (6.2)
Nephrology	13 (5.8)
Haematology	13 (5.8)
Dermatology	12 (5.3)
Others^a^	45 (19.9)
Off-label drug use condition (%)	
Unapproved indication	204 (90.3)
Unapproved condition	10 (4.4)
Unapproved indication and condition	12 (5.3)
Reasons for off-label drug use (%). Lack of clinical response (or suboptimal) to the previous treatments	163 (72.1)
No other drugs approved for that indication/condition	28 (12.4)
Intolerance to the previous treatments	26 (11.5)
Preferred to the alternative drugs for that patient (clinical/logistical reasons)	20 (8.8)
Contraindications to the alternatives	10 (4.4)

^a^ Pneumology (9), rheumatology (9), otorhinolaryngology (6), ophthalmology (4), intensive care medicine (4), thoracic surgery (4), gastrointestinal surgery (3), vascular surgery (2) and other services with only one case (4)

A total of 232 off-label medicines were requested and administered to the 226 patients for 102 different diseases. Two hundred and twenty (97.3 %) patients were treated with one off-label medicine and six (2.7 %) with a combination of two medicines. The most frequent pharmacological subgroups were the monoclonal antibodies (in 56 patients; 24.1 %) and other muscle relaxants (25; 10.8 %). The most frequent medicines were rituximab (49; 21.1 %), botulinum toxin (25; 10.7 %) and omalizumab (14; 6.0 %). Rituximab was used in 22 different indications, botulinum toxin in 5 and omalizumab in 5 (more information on therapeutic subgroups and medicines is available in the annex 1 of the supplementary material). Diseases of the nervous system (31 patients; 13.7 %), neoplasms (30; 13.3 %), diseases of the digestive system (29; 12.8 %) and diseases of the skin and subcutaneous tissue (27; 12 %) were the most frequent conditions. Table 2 shows the most frequent clinical indications in which each off-label medicine was used. Botulinum toxin was used to treat 13 (5.6 %) patients with anal fissure and 8 (3.6 %) with achalasia. Rituximab was used to treat seven (3 %) patients with an acute humoral rejection of a solid organ transplant and six (2.6 %) with pemphigus vulgaris. Omalizumab was used to treat seven patients (3 %) with chronic urticaria.

**Table 2:** Level of evidence-based of most frequently used medicines in each indication

Medicine	Indication	Number (%)	Level of evidence	Ongoing clinical trial
Rituximab	Complications of organ or tissue transplant, failure or rejection	7 (3.0)	4	Phase III
	Pemphigus	6 (2.6)	4	Phase III
	Myasthenia gravis	4 (1.7)	4	Phase II
	Systemic lupus erythematosus (SLE)	4 (1.7)	2b	–
	Cryoglobulinemic purpura	3 (1.3)	2b	Phase II
	Lupus nephritis	3 (1.3)	2b	–
	Wegener granulomatosis	3 (1.3)	1b	Phase III
	Encephalitis, myelitis and encephalomyelitis	2 (0.8)	4	–
	Glomerulonephritis, membranous	2 (0.8)	4	Phase III
	Idiopathic thrombocytopenic purpura	2 (0.8)	1b	Phase III
	Relapsing polychondritis	2 (0.8)	4	–
	Glomerulonephritis, minimal change disease	1 (0.4)	2b	Phase III
	Graft-versus-host disease	1 (0.4)	2a	Phase II
	Lymphoproliferative disorder	1 (0.4)	2b	–
	Neuromyelitis optica	1 (0.4)	4	Phase I
	Polymyositis	1 (0.4)	4	–
	Polyradiculoneuropathy, chronic inflammatory demyelinating	1 (0.4)	4	–
	Sarcoidosis	1 (0.4)	4	Phase II
	Sjögren syndrome	1 (0.4)	2b	Phase II
	Systemic scleroderma	1 (0.4)	2b	Phase II
	Thrombocytopenia in SLE	1 (0.4)	4	–
	Waldenström macroglobulinaemia	1 (0.4)	2b	Phase II
	Subtotal	49 (21.1)		
Botulinum toxin	Anal fissure	13 (5.6)	1a	Phase IV
	Esophageal achalasia	8 (3.4)	1a	–
	Generalized hyperhidrosis	2 (0.8)	1a	Phase IV
	Eyelid retraction	1 (0.4)	2b	Phase IV
	Myofascial pain	1 (0.4)	2b	Phase IV
	Subtotal	25 (10.8)		
Omalizumab	Chronic urticaria	7 (3.0)	2b	Phase III
	Food-induced anaphylaxis	3 (1.3)	4	Phase II
	Cold-induced urticaria	2 (0.8)	4	–
	Extrinsic allergic asthma	1 (0.4)	4	Phase IV
	Nasal polyps	1 (0.4)	4	Phase IV
	Subtotal	14 (6.0)		

Information about the other used medicines is available in annex 2 of the supplementary material

In 117 cases (51.8 %), the level of clinical evidence for using the medicines in the requested conditions was low, and in 109 (48.2 %) was high. The level of evidence was 4 in 107 (47.4 %) cases, 2b in 48 (21.2 %), 1a in 29 (12.8 %), 1b in 23 (10.2 %), 5 in 10 (4.4 %), 1c and 2a each with 4 (1.8 %) and 2c in 1 (0.4 %). There were ongoing clinical trials assessing the efficacy of off-label medicines in 122 cases (54 %), 84 of whom on phases III or IV. Table 2 shows the level of clinical evidence and information about ongoing clinical trials for each pair of clinical conditions and off-label medicines.

In 164 (72.6 %) patients, a clinical response was observed (82 [36.3 %] with a partial clinical response, 71 [31.4 %] with a complete clinical response and 11 [4.9 %] with a stabilization); in 59 patients (26.1 %), a lack of response was documented and in 3 (1.3 %), it was unknown. Patients were concomitantly treated with a median of 2 drugs (IQR 2–4), mainly prednisone (77 cases), metilprednisolone (19), immunoglobulins (13), mycophenolate mofetil (12), tacrolimus (11) and azatioprine (11). Table 3 shows the clinical response to off-label medicines in the different conditions. No statistically significant differences were observed between patients treated with a medicine with a high level of evidence and those treated with medicines with a low level of evidence (76.9 vs. 70.4 %, respectively, *p* = 0,278).

**Table 3:** Outcomes for the most frequently used medicines in each indication

	Complete response (*N*)	Partial response (*N*)	Stabilization (*N*)	No response (*N*)	Total (*N*)
Rituximab					
Complications of organ or tissue transplant, failure or rejection	3	2	–	2	7
Pemphigus	1	4	–	1	6
Myasthenia gravis	2	–	1	1	4
Systemic lupus erythematosus (SLE)	0	2	–	2	4
Cryoglobulinemic purpura	2	1	–	–	3
Lupus nephritis	1	1	–	1	3
Wegener granulomatosis	–	3	–	–	3
Encephalitis, myelitis and encephalomyelitis	–	1	–	1	2
Glomerulonephritis, membranous	–	1	–	1	2
Idiopathic thrombocytopenic purpura	–	–	–	2	2
Relapsing polychondritis	–	–	–	2	2
Glomerulonephritis, minimal change disease	1	–	–	–	1
Graft-versus-host disease	–	1	–	–	1
Lymphoproliferative disorder	1	–	–	–	1
Neuromyelitis optica	–	1	–	–	1
Polymyositis	1	–	–	–	1
Polyradiculoneuropathy, chronic inflammatory demyelinating	–	1	–	–	1
Sarcoidosis	–	–	–	1	1
Sjögren syndrome	–	1	–	–	1
Systemic sclerodermia	–	–	1	–	1
Thrombocytopenia in SLE	1	–	–	–	1
Waldenström macroglobulinaemia	–	1	–	–	1
Subtotal (%)	13 (26.5)	20 (40.8)	2 (4.1)	14 (28.6)	49 (100)
Botulinum toxin					
Anal fissure	6	4	–	3	13
Esophageal achalasia	6	1	–	–	7^a^
Generalized hyperhidrosis	–	1	–	1	2
Eyelid retraction	1	–	–	–	1
Myofascial pain	–	1	–	–	1
Subtotal (%)	13 (54.2)	7 (29.1)	–	4 (16.7)	24 (100)^a^
Omalizumab					
Chronic urticaria	5	1	–	1	7
Food-induced anaphylaxia	2	–	–	1	3
Cold-induced urticaria	–	2	–	–	2
Extrinsic allergic asthma	–	1	–	–	1
Nasal polyps	1	–	–	–	1
Subtotal (%)	8 (57.1)	4 (28.6)	–	2 (14.3)	14 (100)

Information about the other used medicines is available in annex 3 of the supplementary material

^a^One unknown response

A total of 58 (25.7 %) patients experienced 105 adverse effects. The most frequent adverse effects were infections (11 patients; 5.3 %), fatigue (11; 4.9 %), diarrhoea (9; 4 %), rash and other skin disorders (9; 4 %), leukopenia, neutropenia and/or lymphopenia (8; 3.5 %) and nausea and vomiting (5; 2.2 %) and thrombocytopenia (5; 2.2 %). Rituximab, erlotinib and bendamustine were the drugs involved in more adverse effects. In 11 patients (4.9 %), the adverse effects were severe and in 10 patients, this resulted in treatment being withdrawn. In one patient, the adverse effect (varicella pneumonia with rituximab added to other immunosuppressants in a patient with myasthenia gravis) was fatal.

The total cost of off-label medicine treatments was €997,494.71. The median (IQR) cost per patient was €2,943.07 (541.9–5,872.54). The total cost of off-label medicine treatments in clinical conditions with some response was €705,157.35 and for those with no response was €281,626.71. The median cost per patient (IQR) without response was higher (€4,262.8 [594.55–6,770.40]) than that of patients with response (€2,669.01 [449.7–5,463.93]). The total cost of off-label medicines with a high level of evidence was €485,235.89 and for those with a low level was €512,258.82. The median cost per patient (IQR) treated with a medicine with a low level of clinical evidence was higher (€3,085.38 [1,083.76–5,046.81]) than that of patients treated with a medicine with a high level (€2,693.50 [165.48–6,552.0]).

## Discussion

Our study shows that a high percentage of patients treated with off-label medicines had some response, either complete or partial, despite the fact that most of them had failed to respond to several previous treatments. However, one out of four treated patients had adverse events, and the median cost of off-label treatments was relatively high. Although several articles have reported the use of off-label medicines, few of them have assessed outcomes in clinical practice, and most have focused on one specific medicine [12, 18]. We believe this is the first study where the outcomes of patients treated with different off-label medicines have been reported.

It is also interesting to note the wide variety of off-label medicines and indications observed in the study. This high variety has been described previously [22]. New technological medicines such as biological products were frequently used by patients who had severe or life-threatening diseases that had not responded to previous treatments. This is not surprising, given that the study was performed in tertiary hospitals that have highly specialized services. Biologic medicines are being employed more often in clinical practice as off-label treatments in patients with autoimmune diseases and severe clinical symptoms [23]. Rituximab was the most frequently used off-label medicine as has been reported in other studies [12, 13, 18, 22]. In addition, rituximab was used in a lot of different diseases because it is an anti-CD20 monoclonal antibody against B lymphocytes that can be potentially useful in a heterogeneous group of autoimmune and inflammatory diseases. The most frequent indications were transplant-related and dermatological uses, autoimmune tissue and renal diseases. Globally, the responses observed with rituximab were high, but the partial responses predominated. However, variations in the response were observed. In transplant-related issues (mainly humoral acute rejection), more patients with a complete response were identified. In contrast, more patients with non-response were seen in idiopathic thrombocytopenic purpura. Other studies have shown similar outcomes with off-label use of rituximab [12, 18].

Other frequently used medicines were botulinum toxin and omalizumab, but in a smaller range of indications. Botulinum toxin was mainly used in anal fissure and esophageal achalasia. The most prevalent response was a complete response in both diseases. In a systematic review and meta-analysis, botulinum toxin has been similar to glyceryltrinitrate [24] and inferior to the lateral internal sphincterotomy in the management of anal fissure [25]. Omalizumab was often used in chronic spontaneous urticaria, and the responses have been good in most cases. Efficacy of omalizumab in symptomatic patients despite H1-antihistamine therapy has been shown in a clinical trial [26]. Recently, the Committee for Medicinal Products for Human Use (CHMP) from the European Medicines Agency has adopted a positive opinion recommending its use as an add-on therapy to the treatment of chronic urticaria [27].

In our study, the evidence to use these medicines was often low as well as the frequency of ongoing clinical trials assessing their efficacy. In general, the evidence supporting the use of off-label medicines has also been reported as low, although the classifications used to rate the evidence have been quite variable [2, 9, 12, 13, 28]. Moreover, the level of evidence could be influenced by the health area, period of study, kind of medicines and evaluated indications. Thus, on the one hand, Radley et al. described that most off-label medicines used in outpatient care had little or no scientific support [2]. On the other hand, Mellor et al. reported that most off-label anticancer medicines are supported by guidelines or published peer-review research [9]. Future studies should analyse the variability in the level of evidence of off-label medicines according to the different factors mentioned above.

The most frequent reason for off-label use of medicines was for unapproved indications in adults and only a few cases were in children. Our study did not have children as a target population, as opposed to other studies [14–17]. In addition, the use of off-label medicines was identified through the requests for medicines received in pharmacy services. In general, most of these requested medicines are sophisticated and expensive, and these types of medicines are less frequently used in children especially as off-label use.

Risk from medicines is often based on studies performed in approved conditions but limited data are available on safety in unapproved indications. Moreover, patients’ characteristics in unapproved conditions can substantially differ from those of approved indications due to the basal disease state and the immunological situation. Therefore, in case of off-label medicines use, the benefit-risk relation is even more important given the limited available evidence on the efficacy and also safety. Thus, data from the Spanish registry BIOBADASER 2.0 showed a higher frequency of adverse reactions when TNF antagonists were used in unapproved rheumatic conditions than when they were used in approved rheumatic indications [29]. In children, off-label medicines were also more likely to be implicated in an adverse drug reaction than authorized medicines [30]. In our study, around one out of four patients had an adverse reaction related to off-label medicines use and some reactions were severe or life-threatening. Infections followed by gastrointestinal, cutaneous and haematological reactions were the ones most frequently observed. These types of reactions are to be expected bearing in mind how the most frequently administered medicines work.

Another important issue in off-label drug uses is the benefit-cost relation. In our study, the median cost per patient was high because most administered medicines are expensive. However, other studies have shown that the cost of treatment with medicines is higher when they are used for non-approved conditions than for approved indications [31]. Interestingly, in our study, the median cost per patient without response was higher than that of a patient with response, although the difference was not statistically significant. Further studies should analyse the cost of off-label use of medicines compared to the outcomes.

In our study, different medicines were administered for off-label use for a diverse range of clinical conditions and often with a low level of available evidence. Randomized clinical trials should be performed in these conditions but problems in financing and recruiting patients who have rare diseases may make it difficult to conduct them. Alternatively, national or international registers of patients treated with off-label medicines may be useful as a source of information on their effectiveness and safety. In any case, use of these medicines requires a careful assessment of each case and a sensible expectation in relation to clinical outcomes. Le Jeunne et al. proposed a control system for all off-label prescriptions with a dedicated committee which would determine the frame of off-label prescriptions, in order to improve the use of these medicines [32].

Our study has several limitations. Firstly, we did an observational study without a control group of patients and, hence, some biased results could be present in the assessment of clinical outcomes. Secondly, we included a heterogeneous range of diseases and medicines, with few cases in each group followed for a short period of time, and this hinders the analysis and interpretation of results. Thirdly, our study was based on the requests for off-label uses, and this can limit the validity of the study results. Fourthly, the study was performed in five tertiary hospitals in our area, and this limits the extrapolation of results to other hospitals with different characteristics or geographic areas. Nevertheless, the main strength of our study is the assessment of clinical outcomes in different off-label medicines use. Moreover, we have done a multicentric study in large tertiary university hospitals that cover most medical and surgical specialities to a high level of complexity.

In conclusion, in our study, a high variability of off-label medicines and indications was found. Although the clinical evidence of off-label medicines was often low, a high percentage of some clinical responses in patients with previous multiple treatment failures was observed. However, this was at the expenses of adverse effects (some of them severe) and a high cost. Even though more evidence from clinical trials would be desirable, they can be difficult to carry out and finance especially where rare diseases are concerned. Meanwhile, data from observational studies and registers of patients treated with off-label medicines should be kept to obtain information and to assist in prescribing decisions in clinical practice.

## Electronic supplementary material

Below is the link to the electronic supplementary material.

ESM 1 (DOC 618 kb)
