# Supplementary material for: Outcomes of off-label drug uses in hospitals: a multicentric prospective study
Source: Eur J Clin Pharmacol. 2014 Sep 9;70(11):1385–93. doi: 10.1007/s00228-014-1746-2 (PMC4198805; doi:10.1007/s00228-014-1746-2)
Supplement: Supplementary file 1 — (DOC 618 kb) [file 228_2014_1746_MOESM1_ESM.doc]

**Annexed supplementary material**

Annex 1. Therapeutic subgroups and medicines.

| **Therapeutic subgroup** | **N (%)** | **Medicines** | **N (%)** | **Indications N** |
| --- | --- | --- | --- | --- |
| [Monoclonal antibodies](http://www.whocc.no/atc_ddd_index/?code=L01XC) (L01XC) | 56 (24.1) | rituximab | 49 (21.1) | 21 |
|  |  | bevacizumab | 4 (1.7) | 4 |
|  |  | trastuzumab | 2 (0.8) | 2 |
|  |  | ofatumumab | 1 (0.4) | 1 |
| [Other muscle relaxants, peripherally acting agents](http://www.whocc.no/atc_ddd_index/?code=M03AX) (M03AX) | 25 (10.8) | botulinum toxin | 25 (10.8) | 5 |
| [Tumor necrosis factor alpha (TNF-α) inhibitors](http://www.whocc.no/atc_ddd_index/?code=L04AB) (L04AB) | 14 (6.0) | adalimumab | 9 (3.9) | 4 |
|  |  | certolizumab pegol | 2 (0.8) | 1 |
|  |  | infliximab | 2 (0.8) | 2 |
|  |  | etanercept | 1 (0.4) | 1 |
| [Other systemic drugs for obstructive airway diseases](http://www.whocc.no/atc_ddd_index/?code=R03DX) (R03DX) | 14 (6.0) | omalizumab | 14 (6.0) | 5 |
| [Immunoglobulins, normal human](http://www.whocc.no/atc_ddd_index/?code=J06BA) (J06BA) | 11 (4.7) | [immunoglobulins,](http://www.whocc.no/atc_ddd_index/?code=J06BA) | 11 (4.7) | 7 |
| [Interleukin inhibitors](http://www.whocc.no/atc_ddd_index/?code=L04AC) (L04AC) | 10 (4.3) | anakinra | 7 (3.0) | 5 |
|  |  | basiliximab | 1 (0.4) | 1 |
|  |  | tocilizumab | 1 (0.4) | 1 |
|  |  | ustekinumab | 1 (0.4) | 1 |
| [Selective immunosuppressants](http://www.whocc.no/atc_ddd_index/?code=L04AA) (L04AA) | 10 (4.3) | mycophenolic acid | 3 (1.3) | 3 |
|  |  | sirolimus | 3 (1.3) | 3 |
|  |  | eculizumab | 2 (0.8) | 1 |
|  |  | abatacept | 1 (0.4) | 1 |
|  |  | natalizumab | 1 (0.4) | 1 |
| [Protein kinase inhibitors](http://www.whocc.no/atc_ddd_index/?code=L01XE) (L01XE) | 9 (3.9) | erlotinib | 3 (1.3) | 1 |
|  |  | sunitinib | 2 (0.8) | 2 |
|  |  | imatinib | 1 (0.4) | 1 |
|  |  | lapatinib | 1 (0.4) | 1 |
|  |  | pazopanib | 1 (0.4) | 1 |
|  |  | sorafenib | 1 (0.4) | 1 |
| [Other immunosuppressants](http://www.whocc.no/atc_ddd_index/?code=L04AX) (L04AX) | 7 (3.0) | thalidomide | 6 (2.6) | 4 |
|  |  | lenalidomide | 1 (0.4) | 1 |
| [Nucleosides and nucleotides excl. reverse transcriptase inhibitors](http://www.whocc.no/atc_ddd_index/?code=J05AB) (J05AB) | 6 (2.6) | cidifovir | 3 (1.3) | 1 |
|  |  | ganciclovir | 1 (0.4) | 1 |
|  |  | ribavirin | 1 (0.4) | 1 |
|  |  | valganciclovir | 1 (0.4) | 1 |
| [Other antianemic preparations](http://www.whocc.no/atc_ddd_index/?code=B03XA) (B03XA) | 6 (2.6) | erythropoietin | 4 (1.7) | 1 |
|  |  | darbepoetin alfa | 2 (0.8) | 1 |
| [Nitrogen mustard analogues](http://www.whocc.no/atc_ddd_index/?code=L01AA) (L01AA) | 5 (2.1) | bendamustine | 3 (1.3) | 3 |
|  |  | cyclophosphamide | 2 (0.8) | 2 |
| [Other aminoglycosides](http://www.whocc.no/atc_ddd_index/?code=J01GB) (J01GB) | 5 (2.1) | tobramycin | 3 (1.3) | 2 |
|  |  | amikacin | 2 (0.8) | 2 |
| [Drugs used in erectile dysfunction](http://www.whocc.no/atc_ddd_index/?code=G04BE) (G04BE) | 4 (1.7) | sildenafil | 4 (1.7) | 2 |
| [Other antihypertensives](http://www.whocc.no/atc_ddd_index/?code=C02KX) (C02KX) | 4 (1.7) | bosentan | 4 (1.7) | 3 |
| [Other antineoplastic agents](http://www.whocc.no/atc_ddd_index/?code=L01XX) (L01XX) | 4 (1.7) | bortezomib | 2 (0.8) | 2 |
|  |  | irinotecan | 2 (0.8) | 2 |
| [Other antineoplastic agents](http://www.whocc.no/atc_ddd_index/?code=L01XX) (B02BX) | 4 (1.7) | romiplostim | 4 (1.7) | 1 |
| [Other drugs for treatment of tuberculosis](http://www.whocc.no/atc_ddd_index/?code=J04AK) (J04AK**)** | 4 (1.7) | ethambutol | 4 (1.7) | 1 |
| [Bisphosphonates](http://www.whocc.no/atc_ddd_index/?code=M05BA) (M05BA) | 3 (1.3) | pamidronic acid | 3 (1.3) | 3 |
| [Direct thrombin inhibitors](http://www.whocc.no/atc_ddd_index/?code=B01AE) (B01AE) | 3 (1.3) | dabigartran etexilate | 3 (1.3) | 2 |
| [Electrolyte solutions](http://www.whocc.no/atc_ddd_index/?code=B05XA) (B05XA) | 3 (1.3) | potassium phosphate | 3 (1.3) | 1 |
| [Pyrimidine analogues](http://www.whocc.no/atc_ddd_index/?code=L01BC) (L01BC) | 2 (0.8) | azacitidine | 1 (0.4) | 1 |
|  |  | capecitabine | 1 (0.4) | 1 |
| Antidotes (V03AB) | 2 (0.4) | thiosulfate | 1 (0.4) | 1 |
| [Specific immunoglobulins](http://www.whocc.no/atc_ddd_index/?code=J06BB) (J06BB) | 2 (0.8) | palivizumab | 2 (0.8) | 1 |
| Interferons (L03AB) | 2 (0.8) | interferon alpha-2a | 1 (0.4) | 1 |
|  |  | peginterferon alpha-2a | 1 (0.4) | 1 |
| [Other cytotoxic antibiotics](http://www.whocc.no/atc_ddd_index/?code=L01DC) (L01DC) | 2 (0.8) | mitomycin | 2 (0.8) | 2 |
| [Other nervous system drugs](http://www.whocc.no/atc_ddd_index/?code=N07XX) (N07XX) | 2 (0.8) | amifampridine | 1 (0.4) | 1 |
|  |  | fampridine | 1 (0.4) | 1 |
| [Vasopressin antagonists](http://www.whocc.no/atc_ddd_index/?code=C03XA) (C03XA) | 1 (0.4) | tolvaptan | 1 (0.4) | 1 |
| [Second-generation cephalosporins](http://www.whocc.no/atc_ddd_index/?code=J01DC) (J01DC) | 1 (0.4) | cefuroxime | 1 (0.4) | 1 |
| [Glucocorticoids](http://www.whocc.no/atc_ddd_index/?code=H02AB) (H02AB) | 1 (0.4) | triamcinolone | 1 (0.4) | 1 |
| [Nucleoside and nucleotide reverse transcriptase inhibitors](http://www.whocc.no/atc_ddd_index/?code=J05AF) (J05AF) | 1 (0.4) | adefovir dipivoxil | 1 (0.4) | 1 |
| [Calcineurin inhibitors](http://www.whocc.no/atc_ddd_index/?code=L04AD) (L04AD) | 1 (0.4) | tacrolimus | 1 (0.4) | 1 |
| [Other immunostimulants](http://www.whocc.no/atc_ddd_index/?code=L03AX) (L03AX) | 1 (0.4) | glatiramer acetate | 1 (0.4) | 1 |
| [Other alkylating agents](http://www.whocc.no/atc_ddd_index/?code=L01AX) (L01AX) | 1 (0.4) | temozolomide | 1 (0.4) | 1 |
| [Other antiepileptics](http://www.whocc.no/atc_ddd_index/?code=N03AX) (N03AX) | 1 (0.4) | lacosamide | 1 (0.4) | 1 |
| [Other ophthalmologicals](http://www.whocc.no/atc_ddd_index/?code=S01XA) (S01XA) | 1 (0.4) | cyclosporine | 1 (0.4) | 1 |
| [Polymyxins](http://www.whocc.no/atc_ddd_index/?code=J01XB) (J01XB) | 1 (0.4) | colistin | 1 (0.4) | 1 |
| [Propulsives](http://www.whocc.no/atc_ddd_index/?code=A03FA) (A03FA) | 1 (0.4) | cisapride | 1 (0.4) | 1 |
| [Bone morphogenetic proteins](http://www.whocc.no/atc_ddd_index/?code=M05BC) (M05BC) | 1 (0.4) | dibotermin alfa | 1 (0.4) | 1 |
| Taxans (L01CD) | 1 (0.4) | paclitaxel albumin | 1 (0.4) | 1 |

Annex 2. Level of evidence-based of used medicines in each indication

| **Medicine** | **Indication** | **N (%)** | **Level of evidence** | **Ongoing clinical trial** |
| --- | --- | --- | --- | --- |
| Rituximab | Complications of organ or tissue transplant, failure or rejection | 7 (3.0) | 4 | Phase III |
|  | Pemphigus | 6 (2.6) | 4 | Phase III |
|  | Myasthenia gravis | 4 (1.7) | 4 | Phase II |
|  | Systemic lupus erythematosus (SLE) | 4 (1.7) | 2b | - |
|  | Cryoglobulinemic purpura | 3 (1.3) | 2b | Phase II |
|  | Lupus nephritis | 3 (1.3) | 2b | - |
|  | Wegener granulomatosis | 3 (1.3) | 1b | Phase III |
|  | Encephalitis, myelitis and encephalomyelitis | 2 (0.8) | 4 | - |
|  | Glomerulonefritis, membranous | 2 (0.8) | 4 | Phase III |
|  | Idiopathic thrombocytopenic purpura | 2 (0.8) | 1b | Phase III |
|  | Relapsing polychondritis | 2 (0.8) | 4 | - |
|  | Glomerulonephritis, minimal change disease | 1 (0.4) | 2b | Phase III |
|  | Graft-versus-host disease | 1 (0.4) | 2a | Phase II |
|  | Lymphoproliferative disorder | 1 (0.4) | 2b | - |
|  | Neuromyelitis optica | 1 (0.4) | 4 | Phase I |
|  | Polymyositis | 1 (0.4) | 4 | - |
|  | Polyradiculoneuropathy, chronic inflammatory demyelinating | 1 (0.4) | 4 | - |
|  | Sarcoidosis | 1 (0.4) | 4 | Phase II |
|  | Sjögren syndrome | 1 (0.4) | 2b | Phase II |
|  | Systemic scleroderma | 1 (0.4) | 2b | Phase II |
|  | Thrombocytopenia in SLE | 1 (0.4) | 4 | - |
|  | Waldenström macroglobulinaemia | 1 (0.4) | 2b | Phase II |
|  | Subtotal | 49 (21.1) |  |  |
| Botulinum toxin | Anal fissure | 13 (5.6) | 1a | Phase IV |
|  | Esophageal achalasia | 8 (3.4) | 1a | - |
|  | Generalized hyperhidrosis | 2 (0.8) | 1a | Phase IV |
|  | Eyelid retraction | 1 (0.4) | 2b | Phase IV |
|  | Myofascial pain | 1 (0.4) | 2b | Phase IV |
|  | Subtotal | 25 (10.8) |  |  |
| Omalizumab | Chronic urticaria | 7 (3.0) | 2b | Phase III |
|  | Food-induced anaphylaxis | 3 (1.3) | 4 | Phase II |
|  | Cold-induced urticaria | 2 (0.8) | 4 | - |
|  | Extrinsic allergic asthma | 1 (0.4) | 4 | Phase IV |
|  | Nasal polyps | 1 (0.4) | 4 | Phase IV |
|  | Subtotal | 14 (6.0) |  |  |
| Human unspecific immunoglobulins | Encephalitis, myelitis and encephalomyelitis | 4 (1.7) | 4 | - |
|  | Pemphigus | 2 (0.8) | 2b/4^a^ | - |
|  | Cerebellar ataxia | 1 (0.4) | 4 | - |
|  | Myasthenia gravis | 1 (0.4) | 1b | - |
|  | Opsoclonus-myoclonus syndrome | 1 (0.4) | 4 | - |
|  | Stevens-Johnson syndrome | 1 (0.4) | 4 | - |
|  | Thrombocytopenia in SLE | 1 (0.4) | 4 | - |
|  | Subtotal | 11 (4.7) |  |  |
| Adalimumab | Hidradenitis suppurativa | 3 (1.3) | 2 b | Phase III |
|  | Ulcerative colitis | 3 (1.3) | 1b | Phase III |
|  | Chorioretinitis | 2 (0.8) | 4 | Phase III |
|  | Pityriasis rubra pilaris | 1 (0.4) | 4 | - |
|  | Subtotal | 9(3.9) |  |  |
| Anakinra | Familial mediterranean fever | 3 (1.3) | 4 | - |
|  | Erdheim-Chester disease | 1 (0.4) | 4 | - |
|  | Gout | 1 (0.4) | 4 | - |
|  | Juvenile arthritis | 1 (0.4) | 4 | - |
|  | Malignant histiocytosis | 1 (0.4) | 4 | - |
|  | Subtotal | 7(3.0) |  |  |
| Thalidomide | Discoid lupus erythematosus | 3 (1.3) | 4 | - |
|  | Hereditary haemorrhagic telangiectasia | 1 (0.4) | 4 | Phase II |
|  | Multiple myeloma | 1 (0.4) | 4 | - |
|  | Neuroblastoma | 1 (0.4) | 5 | - |
|  | Subtotal | 6 (2.6) |  |  |
| Bevacizumab | Astrocytoma | 1 (0.4) | 4 | Phase II |
|  | Breast cancer, 2^nd^ line | 1 (0.4) | 1b | Phase III |
|  | Glioblastoma | 1 (0.4) | 2b | Phase II |
|  | Retinopathy of prematurity | 1 (0.4) | 2b | Phase III |
|  | Subtotal | 4 (1.7) |  |  |
| Bosentan | Raynaud’s syndrome | 2 (0.8) | 4 | - |
|  | Thromboangiitis obliterans | 1 (0.4) | 4 | Phase II |
|  | Upper extremity thrombosis | 1 (0.4) | 5 | - |
|  | Subtotal | 4 (1.7) |  |  |
| Epoetin alpha | Anaemia secondary to hepatitis C treatment | 4 (1.7) | 1b | Phase IV |
| Ethambutol | Pulmonary tuberculosis^b^ | 4 (1.7) | 1c | - |
| Romiplostim | Secondary thrombocytopenia | 4 (1.7) | 4 | Phase II |
| Sildenafil | Pulmonary arterial hypertension in congenital heart diseases^c^ | 3 (1.3) | 1a | Phase III |
|  | Upper extremity thrombosis | 1 (0.4) | 4 | Phase III |
|  | Subtotal | 4 (1.7) |  |  |
| Bendamustine | Chronic lymphocytic leukemia, 3^rd^ line | 1 (0.4) | 4 | Phase IV |
|  | Hodgkin lymphoma | 1 (0.4) | 4 | Phase II |
|  | Multiple myeloma, 2^nd^ line | 1 (0.4) | 2b | - |
|  | Subtotal | 3 (1.3) |  |  |
| Cidofovir | Laringeal papillomatosis | 3 (1.3) | 4 | Phase III |
| Dabigatran etexilate | Atrial fibrillation^c^ | 2 (0.8) | 1b | Phase III |
|  | Sneddon syndrome | 1 (0.4) | 5 | - |
|  | Subtotal | 3 (1.3) |  |  |
| Erlotinib | Non-small-cell lung cancer, 1^st^ line^c^ | 3 (1.3) | 1a | Phase IV |
| Mycophenolic acid | Crohn disease | 1 (0.4) | 2b | - |
|  | Neuromyelitis optica | 1 (0.4) | 4 | - |
|  | Polyarteritis nodosa | 1 (0.4) | 4 | - |
|  | Subtotal | 3 (1.3) |  |  |
| Pamidronate | Ankylosing spondylitis | 1 (0.4) | 4 | - |
|  | Chronic osteomyelitis | 1 (0.4) | 4 | - |
|  | Calciphylaxis | 1 (0.4) | 4 | . |
|  | Subtotal | 3 (1.3) |  |  |
| Potassium phosphate | Hypophosphatemia^d^ | 3 (1.3) | 5 | - |
| Sirolimus | Chordoma | 1 (0.4) | 4 | - |
|  | Lymphangioleiomyomatosis | 1 (0.4) | 1b | Phase II |
|  | Venous/lymphatic malformation | 1 (0.4) | 4 | Phase II |
|  | Subtotal | 3 (1.3) |  |  |
| Tobramycin | Bronchiectasis, *P. aeruginosa* infection | 2 (0.8) | 2b | - |
|  | Pneumonia, *P. aeruginosa* infection | 1 (0.4) | 4 | - |
|  | Subtotal | 3 (1.3) |  |  |
| Amikacin | Bronchietasis, *Mycobacterium abscessus* infection | 1 (0.4) | 4 | - |
|  | *Klebsiella pneumonie* respiratory infection | 1 (0.4) | 4 | - |
|  | Subtotal | 2 (0.8) |  |  |
| Bortezomib | Complications of organ or tissue transplant, failure or rejection | 1 (0.4) | 4 | - |
|  | Multiple myeloma, 3^rd^ line^c^ | 1 (0.4) | 4 | Phase II |
|  | Subtotal | 2 (0.8) |  |  |
| Certolizumab pegol | Crohn disease | 2 (0.8) | 1b | Phase IV |
| Cyclophosphamide | Encephalitis, myelitis and encephalomyelitis | 1 (0.4) | 2c | - |
|  | Neuromyelitis optica | 1 (0.4) | 4 | - |
|  | Subtotal | 2 (0.8) |  |  |
| Darbepoetin alpha | Anaemia secondary to hepatitis C treatment | 1 (0.4) | 1b | Phase IV |
|  | Microangiopathic haemolytic anaemia | 1 (0.4) | 5 | - |
|  | Subtotal | 2 (0.8) |  |  |
| Eculizumab | Haemolytic uremic syndrome | 2 (0.8) | 4 | Phase II |
| Infliximab | Hidradenitis suppurativa | 1 (0.4) | 2b | - |
|  | Vogt-Koyanagi Syndrome | 1 (0.4) | 4 | - |
|  | Subtotal | 2 (0.8) |  |  |
| Irinotecan | Astrocytoma | 1 (0.4) | 4 | - |
|  | Glioblastoma | 1 (0.4) | 2b | - |
|  | Subtotal | 2 (0.8) |  |  |
| Mytomicyn-C | Conjunctival neoplasia | 1 (0.4) | 4 | - |
|  | Subglotic stenosis | 1 (0.4) | 4 | Phase III |
|  | Subtotal | 2 (0.8) |  |  |
| Palivizumab | Respiratory syncycial virus infection | 2 (0.8) | 2a | - |
| Sodium thiosulphate | Calciphylaxis | 2 (0.8) | 4 | - |
| Sunitinib | Hemangiopericytoma | 1 (0.4) | 2b | - |
|  | Thyroid cancer, medullary, metastatic, 3^rd^ line | 1 (0.4) | 2b | Phase II |
|  | Subtotal | 2 (0.8) |  |  |
| Trastuzumab | Breast cancer, metastatic^c^ | 1 (0.4) | 2a | Phase III |
|  | Lung cancer, metastatic | 1 (0.4) | 2b | - |
|  | Subtotal | 2 (0.8) |  |  |
| Abatacept | Haemolytic uremic syndrome | 1 | 5 | - |
| Adefovir dipivoxil | Hepatitis B virus infection^b^ | 1 (0.4) | 1b | Phase III |
| Azacitidine | Myelodysplastic syndrome, low risk | 1 (0.4) | 2b | Phase II |
| Basiliximab | Complications of organ or tissue transplant, failure or rejection | 1 (0.4) | 1b | - |
| Capecitabine | Pancreas carcinoma, metastatic | 1 (0.4) | 4 | Phase II |
| Cefuroxime | Endophthalmitis | 1 (04) | 4 | - |
| Cyclosporine | Keratitis punctata | 1 (0.4) | 4 | - |
| Cisapride | Gastroparesis | 1 (0.4) | 2b | - |
| Colistimethate sodium | Bronchiectasis, *E.coli* infection, multiresistant | 1 (0.4) | 4 | - |
| 3,4-diaminopyridine | Myasthenia gravis | 1 (0.4) | 2b | Phase III |
| Dibotermine alpha | Femoral pseudoarthrosis | 1 (0.4) | 4 | - |
| Etanercept | Orbital pseudotumor | 1 (0.4) | 4 | - |
| Fampridine | Multiple sclerosis | 1 (0.4) | 1b | Phase II |
| Ganciclovir | Congenital cytomegalovirus infection^b^ | 1 (0.4) | 4 | Phase III |
| Glatiramer acetate | Multiple sclerosis, advanced | 1 (0.4) | 4 | - |
| Imatinib | Erdheim-Chester disease | 1 (0.4) | 4 | - |
| Interferon alpha-2a | Erdheim-Chester disease | 1 (0.4) | 4 | - |
| Lacosamide | Status epilepticus | 1 (04) | 4 | - |
| Lapatinib | Breast cancer, metastatic^c^ | 1 (0.4) | 2a | Phase III |
| Lenalidomide | Primary amyloidosis | 1 (0.4) | 4 | Phase III |
| Nab paclitaxel | Breast cancer, 1^st^ line | 1 (0.4) | 2b | - |
| Natalizumab | Multiple sclerosis, advanced | 1 (0.4) | 4 | - |
| Ofatumumab | Autoimmune neuromuscular disorder | 1 (0.4) | 5 | - |
| Pazopanib | Thyroid cancer, follicular, metastatic | 1 (0.4) | 2b | Phase II |
| Peginterferon alpha-2a | Essential thrombocythemia | 1 (0.4) | 2b | Phase II |
| Ribavirin | Hepatitis E virus infection | 1 (0.4) | 4 | Phase II |
| Sorafenib | Thyroid cancer, medullary | 1 (0.4) | 2b | Phase II |
| Tacrolimus | Chronic urticaria | 1 (0.4) | 4 | - |
| Temozolomide | Neuroendocrine pancreatic cancer, metastatic | 1 (0.4) | 4 | Phase II |
| Tocilizumab | Relapsing polychondritis | 1 (0.4) | 4 | - |
| Tolvantan | Hyponatremia in liver disease | 1 (0.4) | 2b | Phase IV |
| Triamcinolone | Lumbosacral radiculopathy | 1 (0.4) | 4 | - |
| Ustekinnumab | Crohn disease | 1 (0.4) | 2b | Phase III |
| Valganciclovir | Cytomegalovirus infection, treatment | 1 (0.4) | 5 | - |

^a^ Level 4 in one case with rituximab.

^b^ Children aged cases in which the drug was not approved.

^c^ Before its approval in that indication/condition.

^d^ Parenteral formulation administered orally.

Annex 3. Outcomes for the used medicines in each indication

|  | **Complete response**  **N** | **Partial response**  **N** | **Stabilization**  **N** | **No response**  **N** | **Total**  **N** |
| --- | --- | --- | --- | --- | --- |
| **Rituximab** |  |  |  |  |  |
| Complications of organ or tissue transplant, failure or rejection | 3 | 2 | - | 2 | 7 |
| Pemphigus | 1 | 4 |  | 1 | 6 |
| Myasthenia gravis | 2 | - | 1 | 1 | 4 |
| Systemic lupus erythematosus (SLE) | - | 2 | - | 2 | 4 |
| Cryoglobulinemic purpura | 2 (75.0) | 1 | - | - | 3 |
| Lupus nephritis | 1 | 1 | - | 1 | 3 |
| Wegener granulomatosis | - | 3 | - | - | 3 |
| Encephalitis, myelitis and encephalomyelitis | - | 1 | - | 1 | 2 |
| Glomerulonephritis, membranous | - | 1 | - | 1 | 2 |
| Idiopathic thrombocytopenic purpura |  |  |  | 2 | 2 |
| Relapsing polychondritis | - | - | - | 2 | 2 |
| Glomerulonephritis, minimal change disease | 1 | - | - | - | 1 |
| Graft-versus-host disease | - | 1 | - | - | 1 |
| Lymphoproliferative disorder | 1 | - | - | - | 1 |
| Neuromyelitis optica | - | 1 | - | - | 1 |
| Polymyositis | 1 | - | - | - | 1 |
| Polyradiculoneuropathy, chronic inflammatory demyelinating | - | 1 | - | - | 1 |
| Sarcoidosis | - | - | - | 1 | 1 |
| Sjögren syndrome | - | 1 | - | - | 1 |
| Systemic sclerodermia | - | - | 1 | - | 1 |
| Thrombocytopenia in SLE | 1 |  |  |  | 1 |
| Waldenström macroglobulinaemia | - | 1 | - | - | 1 |
| Subtotal (%) | 13 (26.5) | 20 (40.8) | 2 (4.1) | 14 (28.6) | 49 (100) |
| **Botulinum toxin** |  |  |  |  |  |
| Anal fissure | 6 | 4 | - | 3 | 13 |
| Esophageal achalasia | 6 | 1 | - | - | 7 ^a^ |
| Generalized hyperhidrosis | - | 1 | - | 1 | 2 |
| Eyelid retraction | 1 | - | - | - | 1 |
| Myofascial pain | - | 1 | - | - | 1 |
| Subtotal | 13 (54.2) | 7 (29.1) | - | 4 (16.7) | 24 (100)^a^ |
| **Omalizumab** |  |  |  |  |  |
| Chronic urticaria | 5 | 1 | - | 1 | 7 |
| Food-induced anaphylaxia | 2 | - | - | 1 | 3 |
| Cold-induced urticaria | - | 2 | - | - | 2 |
| Extrinsic allergic asthma | - | 1 | - | - | 1 |
| Nasal polyps | 1 | - | - | - | 1 |
| Subtotal (%) | 8 (57.1) | 4 (28.6) | - | 2 (14.3) | 14 (100) |
| **Unspecific human immunoglobulins** |  |  |  |  |  |
| Encephalitis, myelitis and encephalomyelitis | - | 2 | - | 2 | 4 |
| Pemphigus | - | - | - | 2 | 2 |
| Cerebellar ataxia | - | 1 | - | - | 1 |
| Myasthenia gravis | - | 1 | - | - | 1 |
| Opsoclonus-myoclonus syndrome | - | 1 | - | - | 1 |
| Stevens-Johnson syndrome | - | 1 | - | - | 1 |
| Thrombocytopenia in SLE | - | - | - | 1 | 1 |
| Subtotal (%) | - | 6 (54.5) | - | 5 (45.5) | 11 (100) |
| **Adalimumab** |  |  |  |  |  |
| Hidradenitis suppurativa | - | 2 | - | 1 | 3 |
| Ulcerative colitis | - | - | 1 | 2 | 3 |
| Chorioretinitis | - | 2 | - | - | 2 |
| Pityriasis rubra pilaris | - | - | - | 1 | 1 |
| Subtotal (%) | - | 4 (44.4) | 1 (11.2) | 4 (44.4) | 9 (100) |
| **Anakinra** |  |  |  |  |  |
| Familial mediterranean fever | 2 | 1 | - | - | 3 |
| Erdheim-Chester disease | - | 1 | - | - | 1 |
| Gout | 1 | - | - | - | 1 |
| Juvenile arthritis | - | - | - | 1 | 1 |
| Malignant histiocytosis | - | 1 | - | - | 1 |
| Subtotal (%) | 3 (42.9) | 3 (42.9) | - | 1 (14.2) | 7 (100) |
| **Thalidomide** |  |  |  |  |  |
| Discoid lupus erythematosus | 1 | 1 | - | 1 | 3 |
| Hereditary haemorrhagic telangiectasia | - | 1 | - | - | 1 |
| Multiple myeloma | - | 1 | - | - | 1 |
| Neuroblastoma | - | - | 1 | - | 1 |
| Subtotal (%) | 1 (16.7) | 3 (50.0) | 1 (16.7) | 1 (16.7) | 6 (100) |
| **Bevacizumab** |  |  |  |  |  |
| Astrocytoma |  |  |  |  | -^a^ |
| Glioblastoma |  |  |  | 1 | 1 |
| Breast cancer, 2^nd^ line |  | 1 |  |  | 1 |
| Retinopathy of prematurity | 1 |  |  |  | 1 |
| Subtotal (%) | 1 (33.3) | 1 (33.3) |  | 1 (33.3) | 3 (100) |
| **Bosentan** |  |  |  |  |  |
| Raynaud’s syndrome | 1 |  |  | 1 | 2 |
| Thromboangiitis obliterans |  | 1 |  |  | 1 |
| Upper extremity thrombosis |  |  |  | 1 | 1 |
| Subtotal (%) | 1 (25.0) | 1 (25.0) |  | 2 (50.0) | 4 (100) |
| **Epoetin alfa** |  |  |  |  |  |
| Anaemia secondary to hepatitis C treatment |  | 1 | 3 |  | 4 |
| **Ethambutol** |  |  |  |  |  |
| Pulmonary tuberculosis^b^ | 3 | 1 |  |  | 4 |
| **Romiplostim** |  |  |  |  |  |
| Secondary thrombocytopenia |  | 2 |  | 2 | 4 |
| **Sildenafil** |  |  |  |  |  |
| Pulmonary arterial hypertension in congenital heart diseases^c^ |  | 3 |  |  | 3 |
| Upper extremity thrombosis | 1 |  |  |  | 1 |
| Subtotal (%) | 1 (25.0) | 3 (75.0) |  |  | 4 (100) |
| **Bendamustine** |  |  |  |  |  |
| Chronic lymphocytic leukemia, 3^rd^ line |  |  |  | 1 | 1 |
| Hodgkin lymphoma |  | 1 |  |  | 1 |
| Multiple myeloma, 2^nd^ line |  | 1 |  |  | 1 |
| Subtotal (%) |  | 2 |  | 1 | 3 (100) |
| **Cidofovir** |  |  |  |  |  |
| Laringeal papillomatosis | 2 |  |  | 1 | 3 |
| **Dabigatran etexilate** |  |  |  |  |  |
| Atrial fibrillation^c^ |  | 1 | 1 |  | 2 |
| Sneddon syndrome |  | 1 |  |  | 1 |
| Subtotal (%) |  | 2 (66.7) | 1 (33.3) |  | 3 (100) |
| **Erlotinib** |  |  |  |  |  |
| Non-small-cell lung cancer, 1^st^ line |  | 2 |  | 1 | 3 |
| **Mycophenolic acid** |  |  |  |  |  |
| Crohn disease |  |  |  | 1 | 1 |
| Neuromyelitis optica |  | 1 |  |  | 1 |
| Polyarteritis nodosa | 1 |  |  |  | 1 |
| Subtotal (%) | 1 (33.3) | 1 (33.3) |  | 1 (33.3) | 3 (100) |
| **Pamidronate** |  |  |  |  |  |
| Ankylosing spondylitis |  | 1 |  |  | 1 |
| Chronic osteomyelitis |  | 1 |  |  | 1 |
| Calciphylaxis | 1 |  |  |  | 1 |
| Subtotal (%) | 1 (33.3) | 2 (66.7) |  |  | 3 (100) |
| **Potassium phosphate** |  |  |  |  |  |
| Hypophosphatemia^d^ | 1 | 2 |  |  | 3 |
| **Sirolimus** |  |  |  |  |  |
| Chordoma |  |  |  |  | -^a^ |
| Lymphangioleiomyomatosis | 1 |  |  |  | 1 |
| Venous/lymphatic malformation | 1 |  |  |  | 1 |
| Subtotal (%) | 2 (100) |  |  |  | 2 (100)^a^ |
| **Tobramycin** |  |  |  |  |  |
| Bronchiectasis, *P. aeruginosa* infection | 1 | 1 |  |  | 2 |
| Pneumonia, *P. aeruginosa* infection | 1 |  |  |  | 1 |
| Subtotal (%) | 2 (66.7) | 1 (33.3) |  |  | 3 (100) |
| **Amikacin** |  |  |  |  |  |
| Bronchietasis, *Mycobacterium abscessus* infection |  |  |  | 1 | 1 |
| *Klebsiella pneumonie* respiratory infection |  |  |  | 1 | 1 |
| Subtotal (%) |  |  |  | 2 (100) | 2 (100) |
| **Bortezomib** |  |  |  |  |  |
| Complications of organ or tissue transplant, failure or rejection |  |  |  | 1 | 1 |
| Multiple myeloma, 3^rd^ line^c^ |  |  | 1 |  | 1 |
| Subtotal (%) |  |  | 1 (50.0) | 1 (50.0) | 2 (100) |
| **Certolizumab pegol** |  |  |  |  |  |
| Crohn disease |  | 1 |  | 1 | 2 |
| **Cyclophosphamide** |  |  |  |  |  |
| Encephalitis, myelitis and encephalomyelitis |  |  |  | 1 | 1 |
| Neuromyelitis optica |  |  |  | 1 | 1 |
| Subtotal (%) |  |  |  | 2 (100) | 2 (100) |
| **Darbepoetin alpha** |  |  |  |  |  |
| Anaemia secondary to hepatitis C treatment |  |  |  | 1 | 1 |
| Microangiopathic haemolytic anaemia | 1 |  |  |  | 1 |
| Subtotal (%) | 1 (50.0) |  |  | 1 (50.0) | 2 (100) |
| **Eculizumab** |  |  |  |  |  |
| Haemolytic uremic syndrome | 2 |  |  |  | 2 |
| **Infliximab** |  |  |  |  |  |
| Hidradenitis suppurativa |  |  |  | 1 | 1 |
| Vogt-Koyanagi Syndrome |  |  |  | 1 | 1 |
| Subtotal (%) |  |  |  | 2 (100) | 2 (100) |
| **Irinotecan** |  |  |  |  |  |
| Astrocytoma |  |  |  |  | -^a^ |
| Glioblastoma |  |  |  | 1 | 1 |
| Subtotal (%) |  |  |  | 1 (100) | 1 (100)^a^ |
| **Mytomycin C** |  |  |  |  |  |
| Conjunctival neoplasia | 1 |  |  |  | 1 |
| Subglotic stenosis | 1 |  |  |  | 1 |
| Subtotal (%) | 2 (100) |  |  |  | 2 (100) |
| **Palivizumab** |  |  |  |  |  |
| Respiratory syncytial virus infection | 2 |  |  |  | 2 |
| **Sodium thiosulphate** |  |  |  |  |  |
| Calciphylaxis | 2 |  |  |  | 2 |
| **Sunitinib** |  |  |  |  |  |
| Hemangiopericytoma |  |  |  | 1 | 1 |
| Thyroid cancer, medullary, metastatic, 3^rd^ line |  |  |  | 1 | 1 |
| Subtotal (%) |  |  |  | 2 (100) | 2 (100) |
| **Trastuzumab** |  |  |  |  |  |
| Breast cancer, metastatic^c^ |  | 1 |  |  | 1 |
| Lung cancer, metastatic |  |  | 1 |  | 1 |
| Subtotal (%) |  | 1 (50.0) | 1 (50.0) |  | 2 (100) |
| **Abatacept** |  |  |  |  |  |
| Haemolytic uremic syndrome |  |  |  | 1 | 1 |
| **Adefovir dipivoxil** |  |  |  |  |  |
| Hepatitis B virus infection^b^ | 1 |  |  |  | 1 |
| **Azacitidine** |  |  |  |  |  |
| Myelodysplastic syndrome, low risk |  | 1 |  |  | 1 |
| **Basiliximab** |  |  |  |  |  |
| Complications of organ or tissue transplant, failure or rejection |  |  |  | 1 | 1 |
| **Capecitabine** |  |  |  |  |  |
| Pancreas carcinoma, metastatic |  |  |  | 1 | 1 |
| **Cefuroxime** |  |  |  |  |  |
| Endophthalmitis | 1 |  |  |  | 1 |
| **Cyclosporine** |  |  |  |  |  |
| Keratitis punctata |  |  |  | 1 | 1 |
| **Cisapride** |  |  |  |  |  |
| Gastroparesis |  | 1 |  |  | 1 |
| **Colistimethate sodium** |  |  |  |  |  |
| Bronchiectasis, *E. Coli* infection, multiresistant | 1 |  |  |  | 1 |
| **3,4-diaminopyridine** |  |  |  |  |  |
| Myasthenia gravis |  | 1 |  |  | 1 |
| **Dibotermine alpha** |  |  |  |  |  |
| Femoral pseudoarthrosis | 1 |  |  |  | 1 |
| **Etanercept** |  |  |  |  |  |
| Orbital pseudotumor |  |  |  | 1 | 1 |
| **Fampridine** |  |  |  |  |  |
| Multiple sclerosis |  | 1 |  |  | 1 |
| **Ganciclovir** |  |  |  |  |  |
| Congenital cytomegalovirus infection^b^ |  |  |  | 1 | 1 |
| **Glatiramer acetate** |  |  |  |  |  |
| Multiple sclerosis, advanced |  | 1 |  |  | 1 |
| **Imatinib** |  |  |  |  |  |
| Erdheim-Chester disease |  |  |  | 1 | 1 |
| **Interferon alpha-2a** |  |  |  |  |  |
| Erdheim-Chester disease |  | 1 |  |  | 1 |
| **Lacosamide** |  |  |  |  |  |
| Status epilepticus | 1 |  |  |  | 1 |
| **Lapatinib** |  |  |  |  |  |
| Breast cancer, metastatic^c^ |  | 1 |  |  | 1 |
| **Lenalidomide** |  |  |  |  |  |
| Primary amyloidosis |  |  |  | 1 | 1 |
| **Nab paclitaxel** |  |  |  |  |  |
| Breast cancer, 1^st^ line |  |  | 1 |  | 1 |
| **Natalizumab** |  |  |  |  |  |
| Multiple sclerosis, advanced |  | 1 |  |  | 1 |
| **Ofatumumab** |  |  |  |  |  |
| Autoimmune neuromuscular disorder |  | 1 |  |  | 1 |
| **Pazopanib** |  |  |  |  |  |
| Thyroid cancer, follicular, metastatic |  | 1 |  |  | 1 |
| **Peginterferon alpha-2a** |  |  |  |  |  |
| Essential thrombocythemia | 1 |  |  |  | 1 |
| **Ribavirin** |  |  |  |  |  |
| Hepatitis E virus infection | 1 |  |  |  | 1 |
| **Sorafenib** |  |  |  |  |  |
| Thyroid cancer, medullary |  | 1 |  |  | 1 |
| **Tacrolimus** |  |  |  |  |  |
| Chronic urticaria | 1 |  |  |  | 1 |
| **Temozolomide** |  |  |  |  |  |
| Neuroendocrine pancreatic cancer, metastatic |  |  |  | 1 | 1 |
| **Tocilizumab** |  |  |  |  |  |
| Relapsing polychondritis |  |  |  | 1 | 1 |
| **Tolvaptan** |  |  |  |  |  |
| Hyponatremia in liver disease | 1 |  |  |  | 1 |
| **Triamcinolone** |  |  |  |  |  |
| Lumbosacral radiculopathy |  | 1 |  |  | 1 |
| **Ustekinumab** |  |  |  |  |  |
| Crohn disease |  | 1 |  |  | 1 |
| **Valganciclovir** |  |  |  |  |  |
| Cytomegalovirus infection, treatment | 1 |  |  |  | 1 |

^a^ One unknown response.

^b^ Children aged cases in which the drug was not approved.

^c^ Before its approval in that indication/condition.

^d^ Parenteral formulation administered orally.
